# Effect of ligand alkyl chain length on lanthanide extraction using liposomes incorporating diglycolamic acid

**DOI:** 10.1007/s44211-026-00889-y

**Published:** 2026-03-13

**Authors:** Takeru Uehara, Shinya Yamasaki, Sota Shimizu, Yuichi Takaku, Yudai Shigekawa, Aya Sakaguchi

**Affiliations:** Faculty of Pure and Applied Sciences, Center for Research in Radiation, Isotopes, and Earth System Sciences, University of Tsukuba, 1-1-1 Tennodai, Tsukuba, Ibaraki 305-8577 Japan

**Keywords:** Separation, High-level radioactive liquid waste, Diglycolamic acid, Rare earth, Europium, Phospholipid

## Abstract

This study introduces an environmentally benign liposome-based extraction system that selectively separates and recovers lanthanides (Ln) without requiring an organic solvent. Recovery of Ln, particularly from high-level radioactive liquid waste streams, is critical for resource utilization and reducing long-term radiotoxicity. The proposed system incorporates highly Ln-selective diglycolamic acid (DGAA) ligands into phospholipid liposomes. Fluorescence spectroscopy demonstrated that europium (Eu) adsorption, mediated by the hydrophilic head group of the incorporated DGAA ligands, reached equilibrium almost immediately upon mixing. Inductively coupled plasma–mass spectrometry revealed that the adsorption efficiency increased with increasing concentration of incorporated DGAA. Approximately three DGAA ligands are required to adsorb one Eu(III) ion. The selectivity for heavy Ln was comparable to that of conventional DGAA-based solvent-extraction systems and was found to be independent of DGAA alkyl chain length. In contrast, the incorporation stability depended on alkyl chain length; for example, liposomes containing *N*,*N*-didodecyldiglycolamic acid retained the ligand without significant precipitation or fluorescence decrease for 7 days at concentrations up to 237 µM/g-egg lecithin. These findings establish liposome-based extraction as a flexible platform in which the ligand head group governs the adsorption characteristics while the alkyl chain length dictates the incorporation stability, providing a quantitative foundation for future solvent-free separation technologies.

**Graphical abstract:**

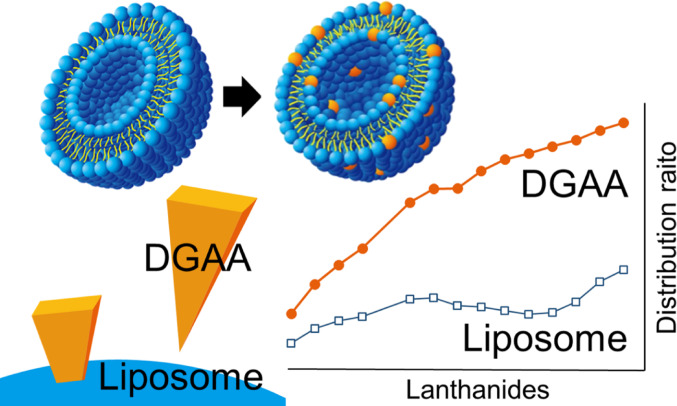

**Supplementary Information:**

The online version contains supplementary material available at 10.1007/s44211-026-00889-y.

## Introduction

Lanthanides (Ln) and other rare-earth elements are of paramount industrial importance, but their supply is constrained by geological distribution problems, difficulties in separation and purification, and environmental concerns, which collectively threaten the global supply chain. To ensure a stable and diversified supply of Ln, industries have pursued various recycling strategies, including Ln recovery from used products and their separation from high-level radioactive liquid waste (HLLW) generated in nuclear power plants [1, 2]. In particular, substantial amounts of Ln are generated as fission products in HLLW from nuclear power plants. Being strong neutron absorbers, Ln must be removed before the transmutation of minor actinides [3]. Therefore, separating and recovering Ln from HLLW is crucial not only for resource reutilization but also for reducing the radiotoxicity of the waste.

Ln separation and recovery is usually performed through solvent extraction [4], a multi-stage process in which an organic solvent containing an extracting ligand is contacted with an aqueous phase. The target element is selectively extracted and then stripped into a fresh aqueous phase. This multi-stage extraction and stripping process recovers the target elements with high purity but requires a large volume of environmentally harmful organic solvents. Alternative methods that avoid the use of organic solvents have been explored in recent years. Operational procedures similar to those of solvent extraction have been reported after replacing organic solvents with ionic liquids [5, 6]. Although ionic liquids are considered safer than organic solvents, their use is limited by high cost. Another solvent-free method is solid-phase extraction, which uses a solid adsorbent with a ligand immobilized on its surface [7–9]. For example, ligands introduced onto a silica gel surface can recover target elements without organic solvents. This method easily recovers the adsorbent particles but the solid phase used for recovery is discarded as low-level radioactive waste after processing HLLW.

**Fig. 1:**
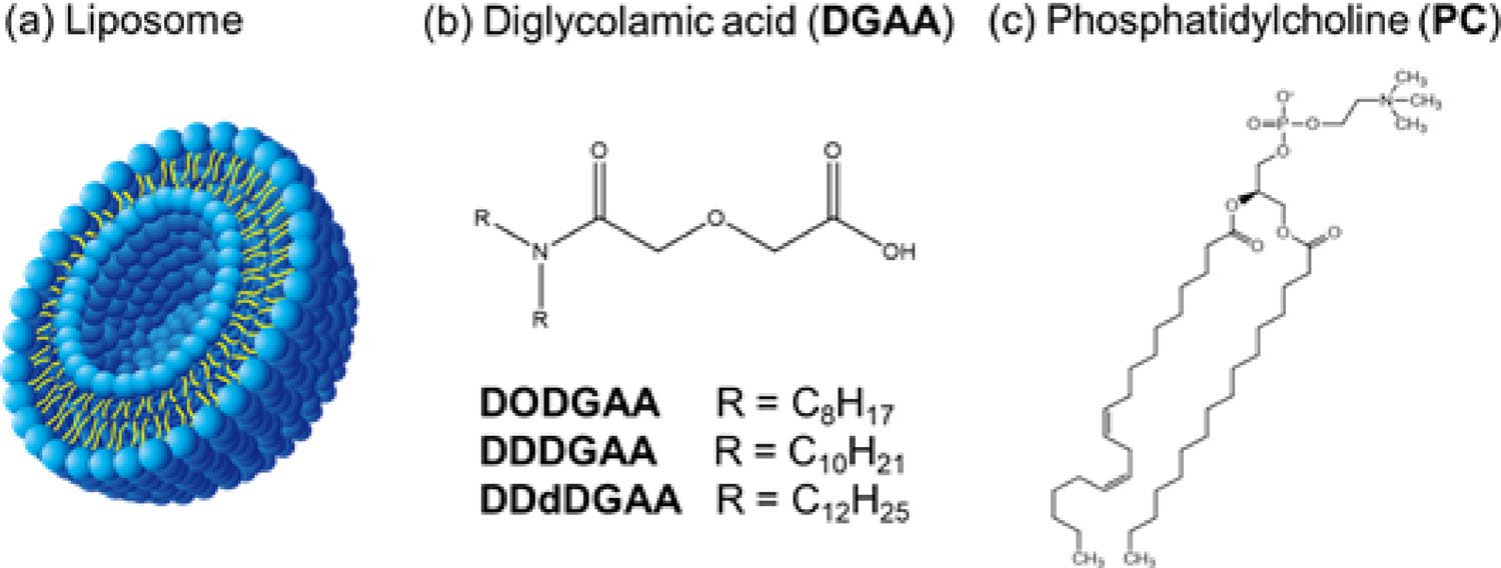
**a** Illustration showing the cross-section of a liposome; **b** Structural formulas of diglycolamic acid; **c** Structural formulas of phosphatidylcholine

This study proposes an extraction system employing liposomes as the extraction medium. Liposomes are spherical bilayer vesicles formed through the self-assembly of phospholipids, which possess an inward-facing hydrophobic part and an outward-facing hydrophilic part (Fig. 1a). As liposomes can incorporate hydrophobic substances within the hydrophobic region of their membrane and water-soluble substances within their internal aqueous compartment, they have been extensively investigated as carriers in drug delivery systems [10, 11]. The present study hypothesizes that solvent-extraction ligands incorporated in the hydrophobic regions of liposomes can recover target ions without requiring large volumes of organic solvents. To ensure its environmental friendliness, this method is designed to minimize secondary waste and maximize volume reduction compared with conventional solvent extraction and solid-phase extraction.

For this initial study, we selected ligands with a diglycolamic acid (DGAA) backbone (Fig. 1b) as model ligands; these are amphiphilic and have been developed for use in conventional solvent extraction [12, 13]. Although DGAA ligands are not suitable for high nitric acid conditions, their application in the present study allows for fundamental studies on their incorporation into liposomes and subsequent Ln adsorption behavior. Analogously to liposome phospholipids such as phosphatidylcholine (PC, Fig. 1c), DGAA lipids possess both hydrophobic and hydrophilic parts. The hydrophobic part of the DGAA ligand will incorporate within the liposome membrane, exposing the hydrophilic part on the surface for adsorption. This system is expected to achieve efficient Ln recovery through complex formation. Liposomes prepared at sizes of several hundred nanometers possess a high surface-to-volume ratio with a large number of accessible reaction sites, which is expected to provide a more rapid system than conventional solid-phase extraction. Furthermore, liposomes sized several hundred nanometers and their adsorbed Ln can be recovered via filtration or salting out. Subsequent incineration yields stable inorganic salts of the target Ln (e.g., LnPO_4_), while minimizing the generation of excess secondary waste.

Several studies have investigated metal ion separation systems in which ligands are incorporated into lipid bilayers [14, 15]. For instance, van Zanten et al. reported Pb^2+^ and Cd^2+^ transport using calixarene derivatives [14], and Gazzaz et al. provided a detailed kinetic analysis of Ni^2+^ complexation using PADA ligands [15]. Similar to the present study, vesicle-based platforms serve as environmentally benign separation media in these systems, thus eliminating the need for organic solvents while achieving rapid extraction rates owing to their high specific surface area. However, most previous studies have focused on model systems, leaving scarce reports on the efficient recovery and separation of industrially critical elements such as Ln. The novelty of this research lies in developing an eco-friendly Ln recovery system that immobilizes DGAA derivatives with high lanthanide selectivity onto liposomal membranes without using organic solvents.

To clarify the adsorption behavior of the liposome-incorporated DGAA ligands, we quantified the Ln concentration using inductively coupled plasma–mass spectrometry (ICP–MS) and analyzed the fluorescence spectra of europium (Eu). We also investigated the effects of three ligands bearing alkyl chains of different lengths on the Ln distribution ratio and the incorporation of these ligands into the liposomes.

## Experimental

### Reagents and materials

Egg lecithin (EL) containing PC as the main component, a phospholipid mixture derived from biological membranes, was obtained from Wako Pure Chemical Industries, Ltd. (Osaka, Japan). *N*,*N*-Dioctyldiglycolamic acid (DODGAA) was obtained from Tokyo Chemical Industry Co., Ltd. (Tokyo, Japan). *N*,*N*-Didecyldiglycolamic acid (DDDGAA) and *N*,*N*-Didodecyldiglycolamic acid (DDdDGAA) were synthesized as reported in the literature [12, 13] (SI. 1). The chemical structures and purity of the synthesized ligands were confirmed through ^1^H NMR spectroscopy and elemental analysis.

Lanthanum(III) nitrate hexahydrate was purchased from FUJIFILM Wako Pure Chemical Corporation. Cerium(III) nitrate hexahydrate, praseodymium(III) nitrate hexahydrate, neodymium(III) nitrate hexahydrate, samarium(III) nitrate hexahydrate, europium(III) nitrate hexahydrate, gadolinium(III) nitrate hexahydrate, terbium(III) nitrate hexahydrate, dysprosium(III) nitrate pentahydrate, and lutetium(III) nitrate tetrahydrate were purchased from Kanto Chemical Co., Inc. (Tokyo, Japan). Holmium(III) nitrate hydrate, erbium(III) nitrate hydrate, and ytterbium(III) nitrate hydrate were purchased from Wako Pure Chemical Industries, Ltd. Thulium(III) nitrate was purchased from Sigma-Aldrich (St. Louis, MO, USA).

The purity of each reagent is provided in the Supplementary Information (Table S1). All reagents were used without further purification. Stock solutions of Ln were prepared by dissolving the corresponding nitrates in nitric acid solutions adjusted to pH 3.0. All solutions were prepared using ultrapure water.

### Preparation of liposome dispersions

EL (0.100 g) and a DGAA ligand (0–356 µmol) were weighed and dissolved in 10 mL of chloroform. The DGAA concentration was defined in terms of two metrics based on the weighed mass of DGAA: (1) specific loading on the EL substrate (µmol/g-EL), analogous to the functional group density in a resin, and (2) the homogeneous aqueous concentration (µM) assuming a uniform distribution. The solution was transferred to a 100-mL round-bottom flask and the solvent was evaporated using a rotary evaporator (Buchi; Flawil, Switzerland) at 318 hPa in a water bath maintained at 50℃ and a cooling water temperature below 5℃ for 2 h, obtaining a thin film. The film was further dried in a desiccator for over 24 h. After adding 10 mL of ultrapure water to the dried film, the mixture was vortexed to obtain a suspension (FLX-S, Front Lab; Tokyo, Japan). The suspension was transferred to a 15-mL centrifuge tube and sonicated using an ultrasonic homogenizer (THU-80, AS ONE; Osaka, Japan) at minimum power for 5 min, yielding a liposome dispersion. The dispersion was syringe-filtered through a 0.20-µm pore size membrane filter (mixed cellulose ester, ADVANTEC; Tokyo, Japan) immediately before use. Unless otherwise stated, the liposome dispersions were used within 1 h after syringe filtration. The particle-size distribution (Figs. S1–S3) and zeta potential (Table S2) were evaluated via dynamic light scattering and electrophoretic light scattering, respectively, using a ZETASIZER NANO-ZS (Malvern Panalytical; Malvern, United Kingdom).

## Adsorption on liposomes incorporating DGAA ligands

### Effect of ligand concentration

To confirm the extraction functionality of the liposome-incorporated DGAA, the changes in Eu fluorescence spectra and Eu adsorption amounts were investigated as functions of DODGAA ligand loading. For this purpose, liposome dispersions were prepared with DODGAA concentrations ranging from 19 to 293 µmol/g-EL.

For fluorescence measurements, the prepared liposome dispersions were mixed with an Eu solution. Each mixture was then diluted to final concentrations of 1.00 mg/g EL and 330 µM Eu with nitric acid solution (pH 3.0). This dispersion was stirred for 15 min in a mix rotator (VMRC-5, AS ONE) at 20 rpm at 298 K, and its fluorescence spectrum was then acquired.

To quantify the adsorption, a similar mixture was diluted to final concentrations of 0.100 mg/g EL and 33.0 µM Eu and stirred for 15 min. This dispersion was vacuum-filtered through a 0.025-µm pore size membrane filter (mixed cellulose ester, Merck; Darmstadt, Germany) to remove the liposomes and adsorbed Eu. The Eu concentration in the resulting filtrate was quantified using ICP–MS.

### Effect of pH

To investigate the pH dependence of Eu adsorption by the liposome-incorporated DGAA, the adsorption amounts were compared under different pH conditions. Liposome dispersions with a fixed DODGAA concentration (111 µmol/g-EL) were mixed with an Eu solution and the resulting mixture was diluted to final concentrations of 0.100 mg/g EL and 61.0 µM Eu in nitric acid solution (pH 1.0–5.0). After stirring for 15 min, the dispersion was vacuum-filtered and the Eu concentration in the filtrate was quantified using ICP–MS.

### Effect of reaction time

To determine the time required to reach equilibrium adsorption, the temporal variation of the Eu fluorescence spectrum was monitored after mixing liposome dispersions prepared at a DODGAA concentration of 111 µmol/g-EL with an Eu solution. This mixture was then diluted with nitric acid solution (pH 3.0) to final concentrations of 1.00 mg/g EL and 330 µM Eu. Sixty fluorescence measurements were collected at 46-s intervals, starting at 1 min after dilution. The time required to reach equilibrium was determined from the results.

### Effect of DGAA alkyl chains on adsorption

To investigate the effect of DGAA alkyl chain on the Eu extraction behavior, the Eu adsorption amounts and Ln distribution ratios were compared among three ligands: DODGAA, DDDGAA, and DDdDGAA.

For the adsorption isotherm comparison, liposome dispersions with different DGAA concentrations (12–218 µmol/g-EL) were mixed with an Eu solution. Each mixture was diluted to final concentrations of 0.100 mg/g EL and 33.0 µM Eu with nitric acid solution (pH 3.0) and then stirred for 15 min. After vacuum-filtering the resulting dispersion, the Eu concentration in the filtrate was quantified using ICP–MS.

For the selectivity comparison, liposome dispersions with a fixed DGAA concentration (119 µmol/g-EL) were mixed with a solution containing all Ln elements except promethium. The mixtures were processed as described for the adsorption isotherm experiments: dilution to 0.100 mg/g EL and 4.0 µM for each Ln, stirring for 15 min, vacuum filtration, and ICP–MS-based quantification of Ln concentration in the filtrate.

### Effect of DGAA alkyl chains on liposome incorporation stability

The stability of the liposome-incorporated DGAA ligands was monitored through the changes in adsorption performance during long-term storage. Liposome dispersion containing each of the three ligands (DODGAA, DDDGAA, and DDdDGAA) were prepared at DGAA concentrations ranging from 30 to 356 µmol/g-EL and stored at 4℃.

Immediately after preparation, after 1 day, and after 7 days, the samples were collected and syringe-filtered through a 0.2-µm pore size membrane filter. Each filtered liposome dispersion was mixed with an Eu solution and diluted to final concentrations of 1.00 mg/g EL and 330 µM Eu with nitric acid solution (pH 3.0). The resulting mixtures were stirred for 15 min and then subjected to fluorescence measurements.

### Eu fluorescence spectroscopy

The Eu fluorescence spectrum of the post-adsorption dispersion was obtained on a spectrofluorometer (FP-6500, JASCO; Tokyo, Japan). As the raw data included a scattering background signal originating from EL (1.00 mg/g), the data were fitted to a fourth-order polynomial function and corrected to obtain the final Eu fluorescence spectrum.

### Quantification of adsorption amount

After removing the liposomes and their adsorbed Ln by vacuum filtration, the residual Ln concentration in the filtrate was quantified using ICP–MS (Agilent 7700, Agilent technologies; Santa Clara, CA, USA). The concentration of Ln absorbed on the DGAA-incorporated liposomes, [Ln]_abs_, was calculated as follows:

$$\:{\left[\mathbf{L}\mathbf{n}\right]}_{\mathbf{a}\mathbf{b}\mathbf{s}}\:={\left[\mathbf{L}\mathbf{n}\right]}_{\mathbf{i}\mathbf{n}\mathbf{i}\mathbf{t}\mathbf{i}\mathbf{a}\mathbf{l}}-{\left[\mathbf{L}\mathbf{n}\right]}_{\mathbf{f}\mathbf{i}\mathbf{l}\mathbf{t}\mathbf{r}\mathbf{a}\mathbf{t}\mathbf{e}}\:$$

where [Ln]_initial_ and [Ln]_filtrate_ denote the initial and filtrate Ln concentrations, respectively, determined by ICP–MS. The initial Ln concentration was determined in a reference solution, in which the liposome component was substituted with a nitric acid solution at the appropriate pH without prior filtration. The distribution ratio *D* of Ln was defined as follows:

$$\:D=\frac{{\left[\mathrm{L}\mathrm{n}\right]}_{\mathrm{a}\mathrm{b}\mathrm{s}}}{{\left[\mathrm{L}\mathrm{n}\right]}_{\mathrm{f}\mathrm{i}\mathrm{l}\mathrm{t}\mathrm{r}\mathrm{a}\mathrm{t}\mathrm{e}}}$$

## Results and discussion

### Eu adsorption on the liposome incorporating DODGAA ligand

#### Effect of ligand concentration

**Fig. 2:**
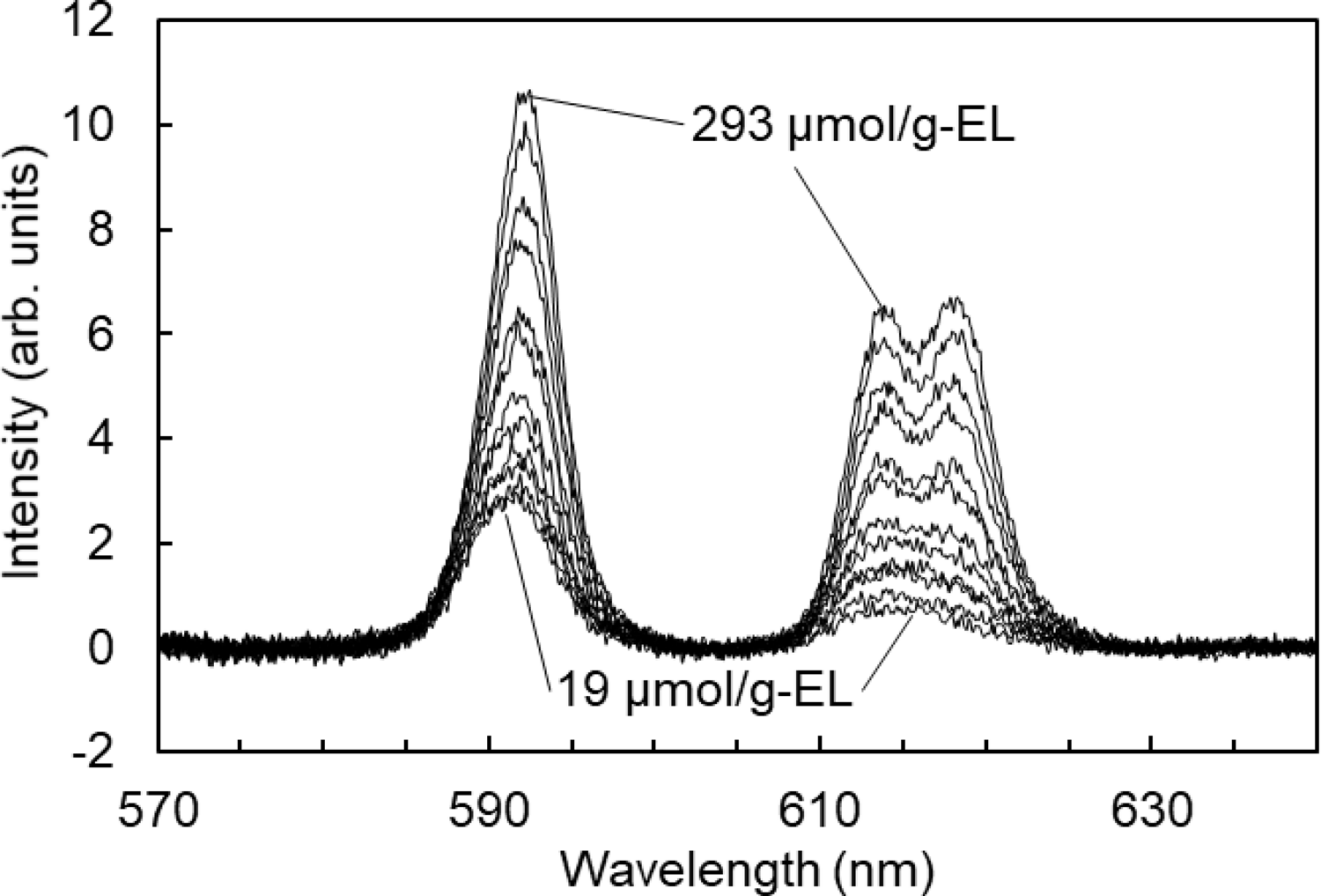
Fluorescence spectra of Eu^3+^ in the liposome incorporating DODGAA ligand dispersion. [DODGAA] = 19–293 µmol/g-EL; [EL] = 1.00 mg/g; [Eu] = 330 µM; pH = 3.0; stirring time = 15 min; excitation bandwidth = 3 nm; fluorescence bandwidth = 3 nm; excitation wavelength = 394 nm. DODGAA = dioctyldiglycolamic acid; EL = egg lecithin

The fluorescence spectra of the Eu–liposome complexes revealed the effectiveness of Ln adsorption on the liposome-incorporated DODGAA (Fig. 2). The fluorescence peaks at 593 and 616 nm are the characteristic wavelengths of the ^5^D_0_→^7^F_1_ and ^5^D_0_→^7^F_2_ emission transitions of Eu^3+^, respectively. As the ^5^D_0_→^7^F_2_ transition is allowed by electric dipoles, it is sensitive to loss of centrosymmetry around Eu [15, 16]. The increasing degree of peak-splitting and intensity of each peak with increasing ligand concentration reflects the increasing degree of coordination of DODGAA ligands to Eu³⁺ ions. Meanwhile, the ^5^D_0_→^7^F_1_ transition is allowed by magnetic dipoles and is typically insensitive to symmetry. The increasing intensity of the 593-nm peak with increasing ligand concentration is likely attributable to suppression of non-radiative deactivation as the coordinated water molecules are increasingly substituted by DGAA ligands, thereby enhancing the fluorescence quantum yield.

**Fig. 3:**
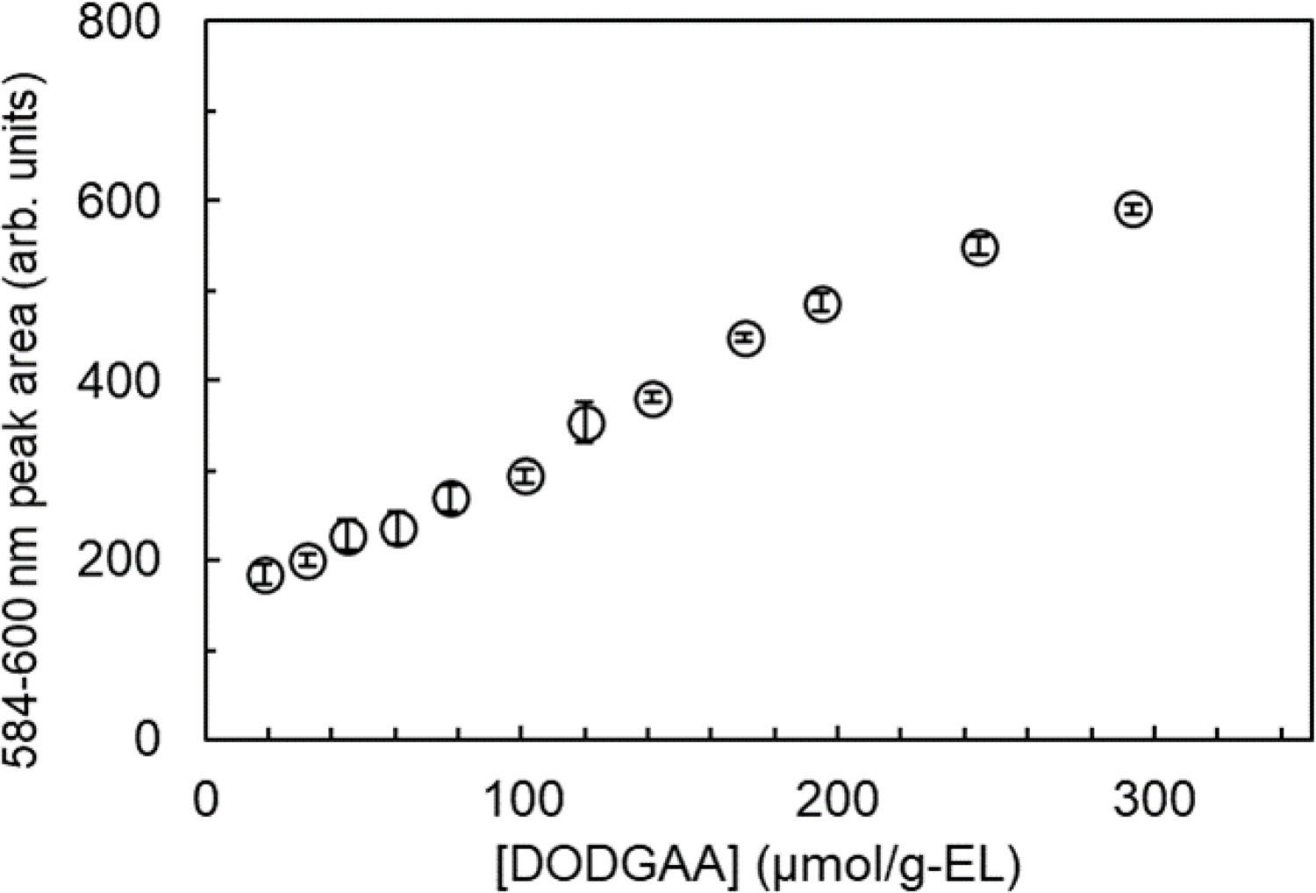
Area of the 584–600 nm fluorescence peak from Fig. 2 versus DODGAA concentration. [EL] = 1.00 mg/g; [Eu] = 330 µM; pH = 3.0; stirring time = 15 min; *n* = 3 (error bars show two standard deviations)

**Fig. 4:**
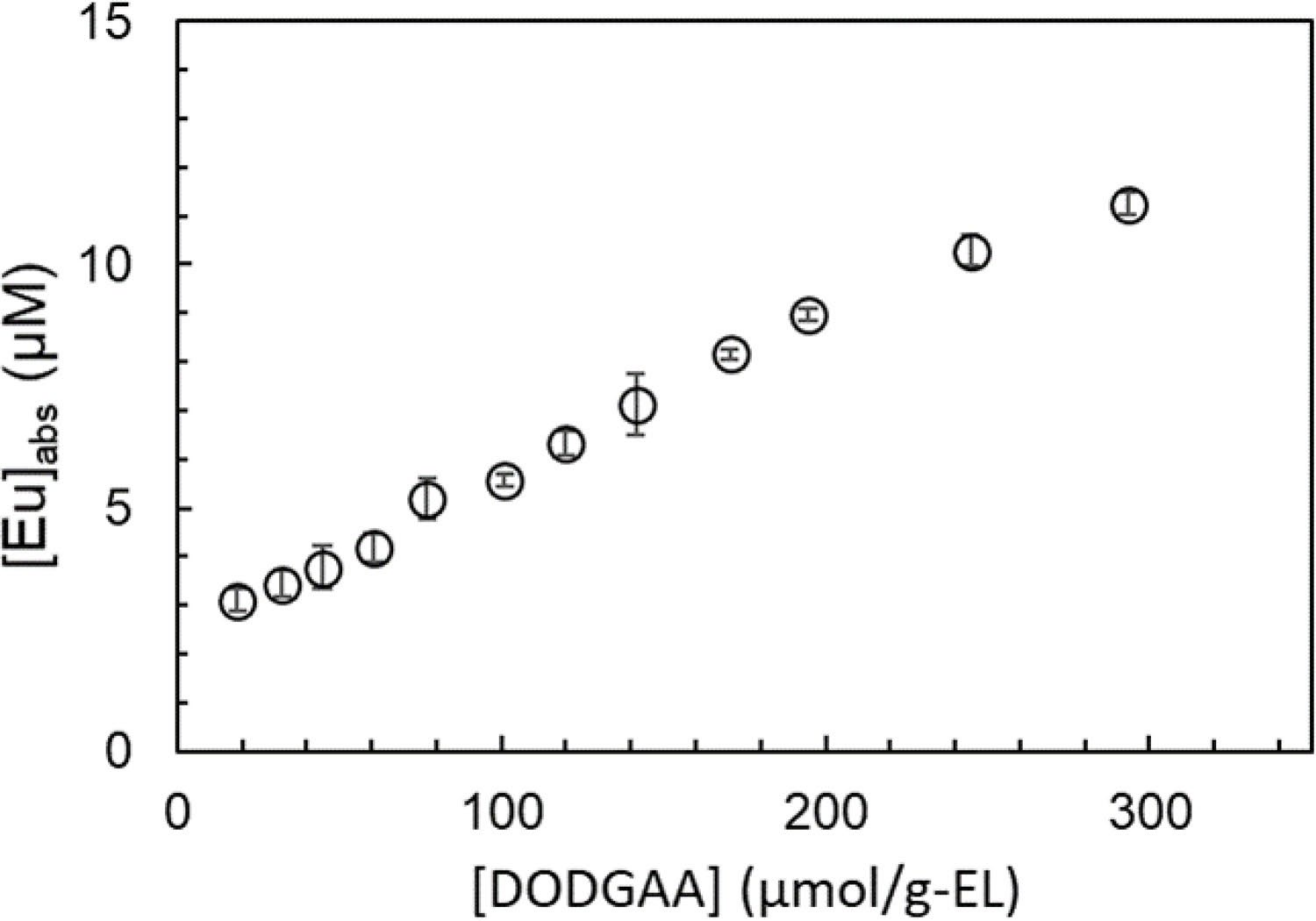
Concentration of Eu adsorbed on DODGAA-incorporated liposomes versus DODGAA concentration. [EL] = 0.100 mg/g; [Eu] = 33.0 µM; pH = 3.0; stirring time = 15 min; *n* = 3 (error bars show two standard deviation)

Figures 3 and 4 plot the area under the 584–600 nm (^5^D_0_→^7^F_1_) peak (centered at 593 nm) and the concentration of Eu absorbed on liposomes incorporating DODGAA ligand, respectively, as functions of DODGAA concentration. The latter was quantified from ICP–MS determinations of Eu concentration in the filtrate. In both plots, the Eu absorbed on the liposomes incorporating DODGAA ligand increased linearly with DODGAA concentration, confirming that Eu adsorption is mediated by the liposome-incorporated DODGAA. The adsorbed Eu amount determined by ICP–MS was strongly linearly correlated with the area of the 584–600-nm fluorescence peak (*R* = 0.998; Fig. S4). Therefore, in subsequent experiments, the fluorescence peak area at 584–600 nm was treated as qualitatively equivalent to the amount of adsorbed Eu.

### Effect of pH

**Fig. 5:**
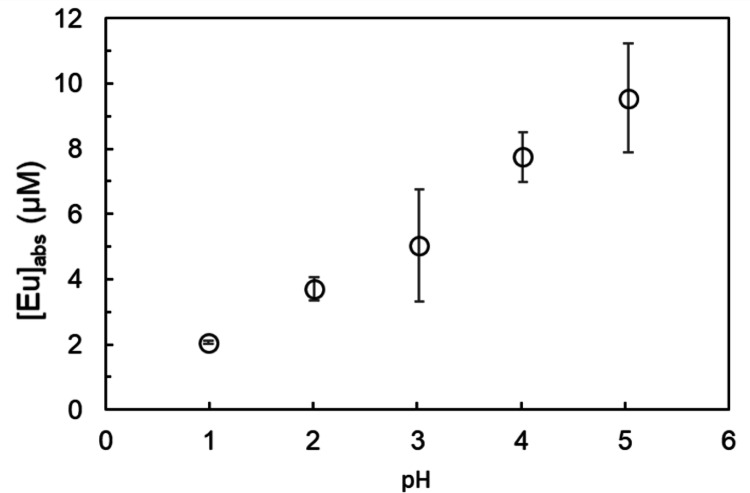
Eu concentration absorbed on the DODGAA-incorporated liposomes versus pH. [DODGAA] = 111 µmol/g-EL; [EL] = 0.100 mg/g; [Eu] = 61.0 µM; stirring time: 15 min; *n* = 3 (error bars show two standard deviation)

DGAA ligands are recognized for their efficient Ln absorption at high pH [12, 13, 17]. The adsorption of Ln to EL also increases with increasing pH [18]. As shown in Fig. 5, Eu adsorption to DGAA increased as the pH increased from 1 to 5. The adsorption behavior of Eu^3+^ at pH 3 is governed by a competitive equilibrium involving the protonated DGAA ligand and interactions with the EL liposome surface. According to the reported pKa value of 5 for DGAA [19], we can quantitatively assess the dominant species at pH 3 using the following expression: [A^−^]/[HA] = 0.01.

This calculation indicates that approximately 99% of the DGAA carboxyl groups are in the protonated form (HA) at pH 3, i.e., the deprotonated carboxylate anion (A^−^) is the minority species in the initial solution. However, Fig. 5 clearly shows that substantial Eu^3+^ adsorption occurs even at pH 3, with the adsorption amount increasing with pH. This result can be explained by the competitive equilibrium between Eu^3+^ and H^+^ for binding to the DGAA ligand. As Eu^3+^ coordinates with A^−^, the equilibrium shifts, causing further deprotonation of HA and continued Eu^3+^ uptake.

Furthermore, prior studies have shown that Ln adsorption on EL liposomes without DGAA increases in the pH range of 3–5 [18]. Therefore, the overall increase in adsorption from pH 3 to 5 (Fig. 5) is attributed to the combined effect of the pH-dependent competitive binding of Eu^3+^ to the DGAA ligand (where the A^−^ fraction increases with pH) and the nonspecific electrostatic interactions with the PC-based EL liposome surface. Therefore, to minimize the variability, subsequent adsorption experiments were conducted at pH 3.0.

Although the current DGAA ligands are not suitable for direct application to HLLW streams owing to limited adsorption under highly acidic conditions, these results demonstrate their potential applicability for Ln recovery from less acidic industrial wastewater or urban mines.

### Effect of reaction time

**Fig. 6:**
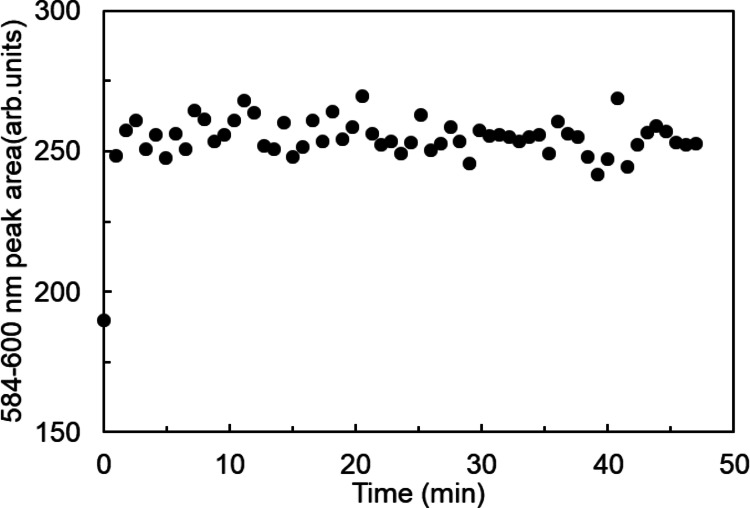
Time dependence of fluorescence peak area. [DODGAA] = 111 µmol/g-EL; [EL] = 1.00 mg/g; [Eu] = 330 µM; pH = 3.0. The data at *t* = 0 were collected from liposome-free Eu solutions at the same concentration

The adsorption kinetics were monitored through their fluorescence changes. As shown in Fig. 6, the fluorescence intensity increased immediately after mixing and reached equilibrium within 1 min. This rapid equilibration is kinetically advantageous over conventional solid-phase extraction systems. For instance, previous studies reported that polymerized resins [7] and impregnated resins [9] require more than 2 h and 500 s, respectively, to reach equilibrium. This rapid equilibrium suggests that Eu^3+^ efficiently interacted with the hydrophilic head groups presented on the liposome surface. The fluorescence signal remained stable for at least two days (298 K), confirming the long-term stability of the interaction. In subsequent experiments, the stirring time was fixed at 15 min.

### Effect of DGAA alkyl chain length on eu adsorption

#### Comparison of adsorption reaction ratios

**Fig. 7:**
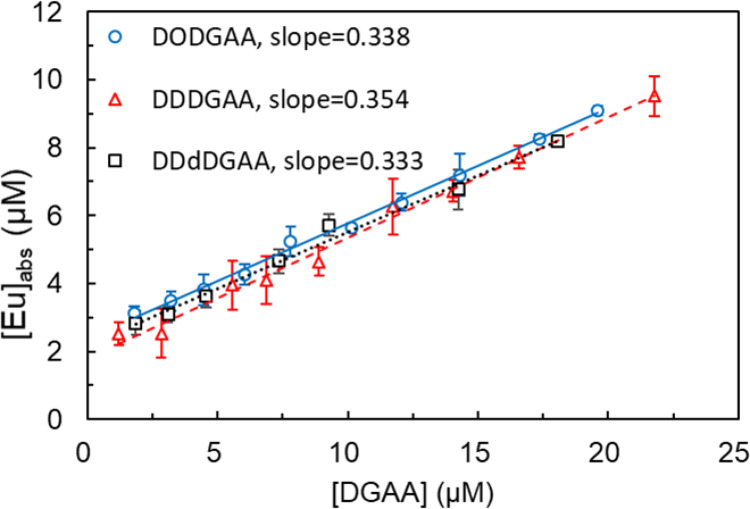
Concentration of Eu absorbed on the DGAA-incorporated liposomes versus DGAA concentration [EL] = 0.100 mg/g; [Eu] = 33.0 µM; pH = 3.0; stirring time = 15 min; *n* = 3 (error bars show two standard deviations). DGAA = diglycolamic acid; DDDGA = *N*,*N*-didecyldiglycolamic acid; DDdGAA = *N*,*N*-didodecyldiglycolamic acid

Having established the role of liposome-incorporated DODGAA in Eu adsorption, we compared the Eu adsorption efficacy of ligands with different alkyl chain lengths (DODGAA, DDDGAA, and DDdDGAA). The Eu adsorption on all three ligands linearly increased with increasing ligand concentration (Fig. 7). The linear regression lines of all ligands yielded similar slopes (~ 0.34) and *y*-intercepts (~ 2 µM, corresponding to Eu–EL adsorption [20]).

In this liposomal system, the amount of ligands that can be incorporated is physically limited, resulting in a low ligand-to-metal concentration ratio. Under such conditions, the stoichiometric ratio can be directly estimated from the slope of the adsorption plot (Δ[Eu]_adsorbed_/Δ[Ligand]_added_) rather than using a conventional equilibrium-based slope analysis with distribution ratio. The obtained slope of approximately 0.34 (~ 1/3) indicates that the adsorption of one Eu³⁺ ion consumes three DGAA ligands, suggesting a 1:3 (Eu: DGAA) stoichiometric composition. This finding agrees well with literature reports for similar systems; however, future work is needed to definitively determine the coordination number of the complex using more rigorous methods, such as NMR titration or ITC.

As the adsorption behaviors of the three ligands showed no significant differences, it was concluded that Eu adsorption is independent of alkyl chain length and is governed solely by the hydrophilic head group.

This stoichiometric efficiency, achieved at a phospholipid concentration of 0.1 mg/g (see the Experimental section), directly translates into environmental advantages for the system. Under these conditions, the phosphorus-to-Eu molar ratio is approximately 13:1. This ratio is remarkably low compared with that in conventional solvent extraction, where the organic phase volume often exceeds the metal mass by several orders of magnitude. Furthermore, unlike solid-phase extraction, relaying on bulky solid supports and often requiring elution steps that generate additional liquid waste, our system enables direct incineration of the medium. As the organic components (C, H, and O) are removed as gases during incineration, phosphorus reacts with Eu to form a stable, insoluble phosphate matrix (LnPO_4_). Thus, the system achieves remarkable volume reduction and integrated waste stabilization, minimizing secondary waste generation compared with solvent extraction and solid-phase extraction.

### Comparison of Ln distribution ratios

**Fig. 8:**
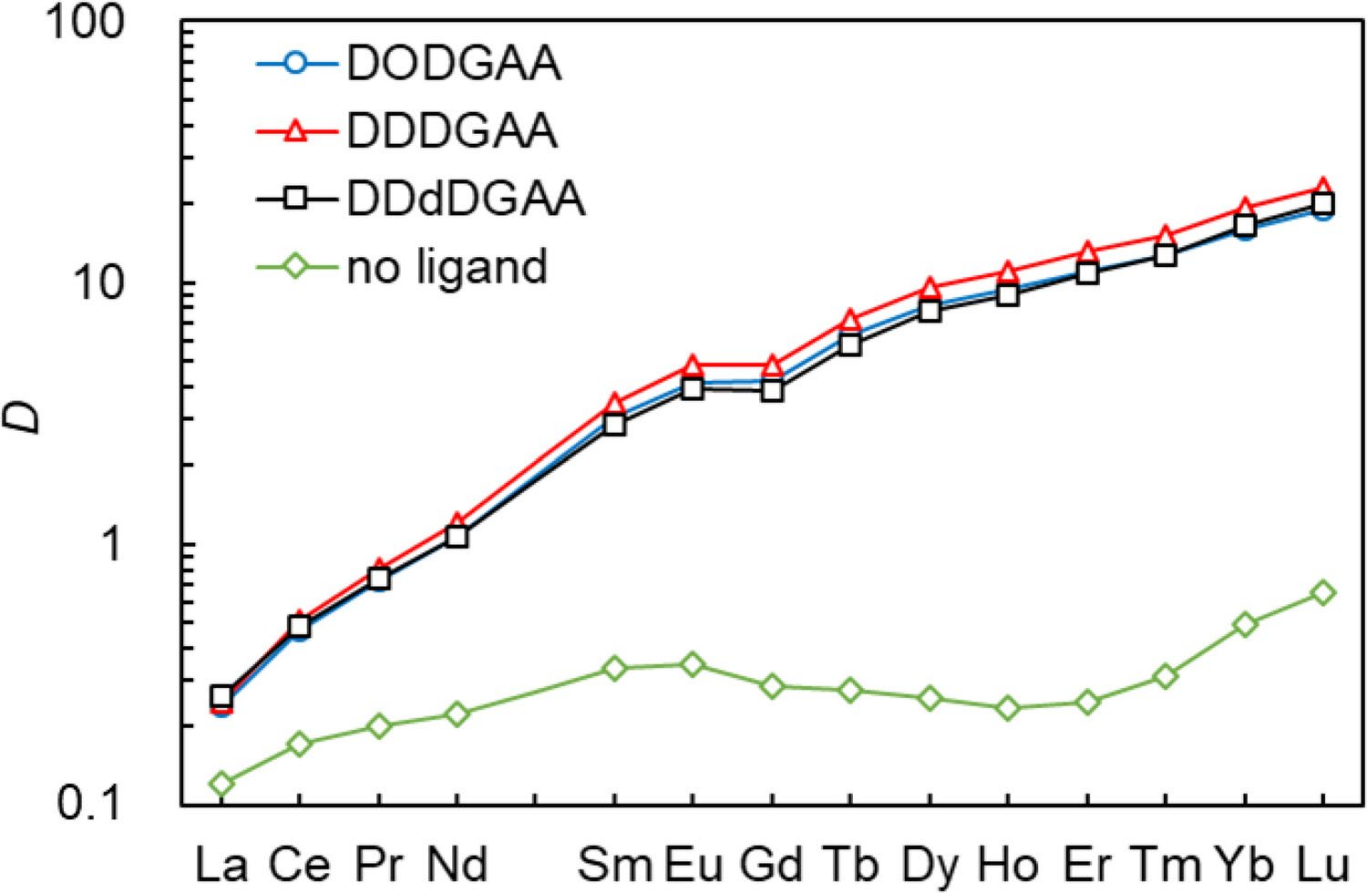
Distribution ratios of Ln; [DODGAA] = 121 µmol/g-EL; [DDDGAA] = 124 µmol/g-EL; [DDdDGAA] = 119 µmol/g-EL; [EL] = 0.100 mg/g; [Ln]_each_ = 4.0 µM; pH = 3.0; stirring time = 15 min

To further examine the chain length effects, liposomes containing equivalent molar amounts of each DGAA ligand were exposed to an Ln mixed solution and their distribution ratios were obtained from the ICP–MS-obtained Ln concentrations (Fig. 8). The distribution ratios and heavy Ln selectivities were higher on all DGAA-incorporated liposomes than on the EL-only liposomes. No significant differences were observed among the ligands, reconfirming that Ln adsorption is governed mainly by the ligand head group rather than by the alkyl chain length. The observation that the adsorption behavior is solely determined by the hydrophilic head group, irrespective of the hydrophobic alkyl chain length, provides a clear path for future ligand design. By appropriately modifying the hydrophilic head group structure—for example, by introducing functional groups effective at high nitric acid concentrations—the system could be adapted for HLLW processing.

### Effect of DGAA alkyl chain length on liposome incorporation

The stabilities of the liposome-incorporated DGAA ligands were determined from the temporal variations in adsorption capacities of the liposome preparations.

**Fig. 9:**
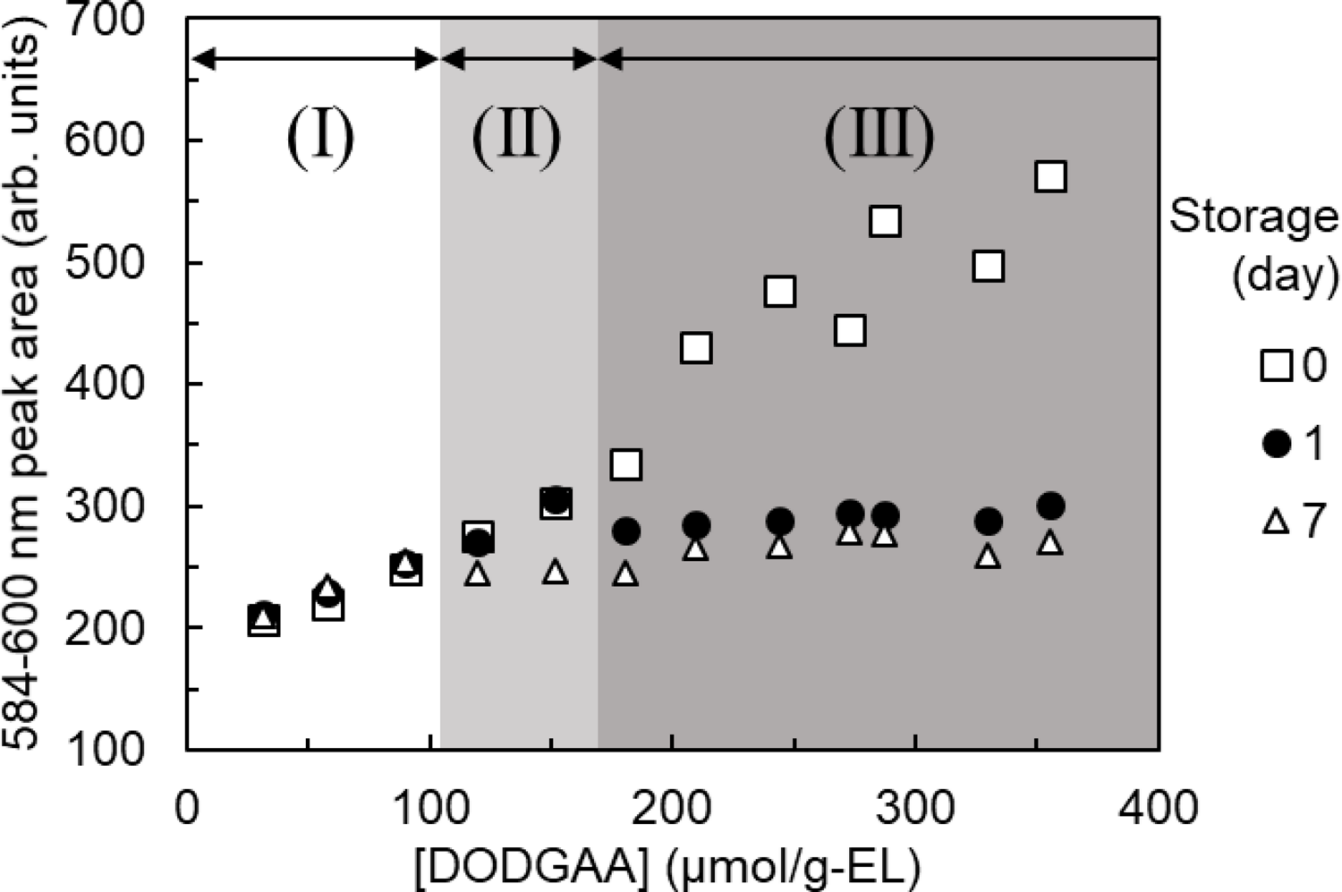
Changes in fluorescence peak area versus DODGAA concentration at different storage times. [EL] = 1.00 mg/g; [Eu] = 330 µM; pH: 3.0; Stirring time = 15 min. The shaded areas labeled (I), (II), and (III) represent the Long-Term Stability Region, Short-Term Stability Region, and Unstable Region, respectively

**Fig. 10:**
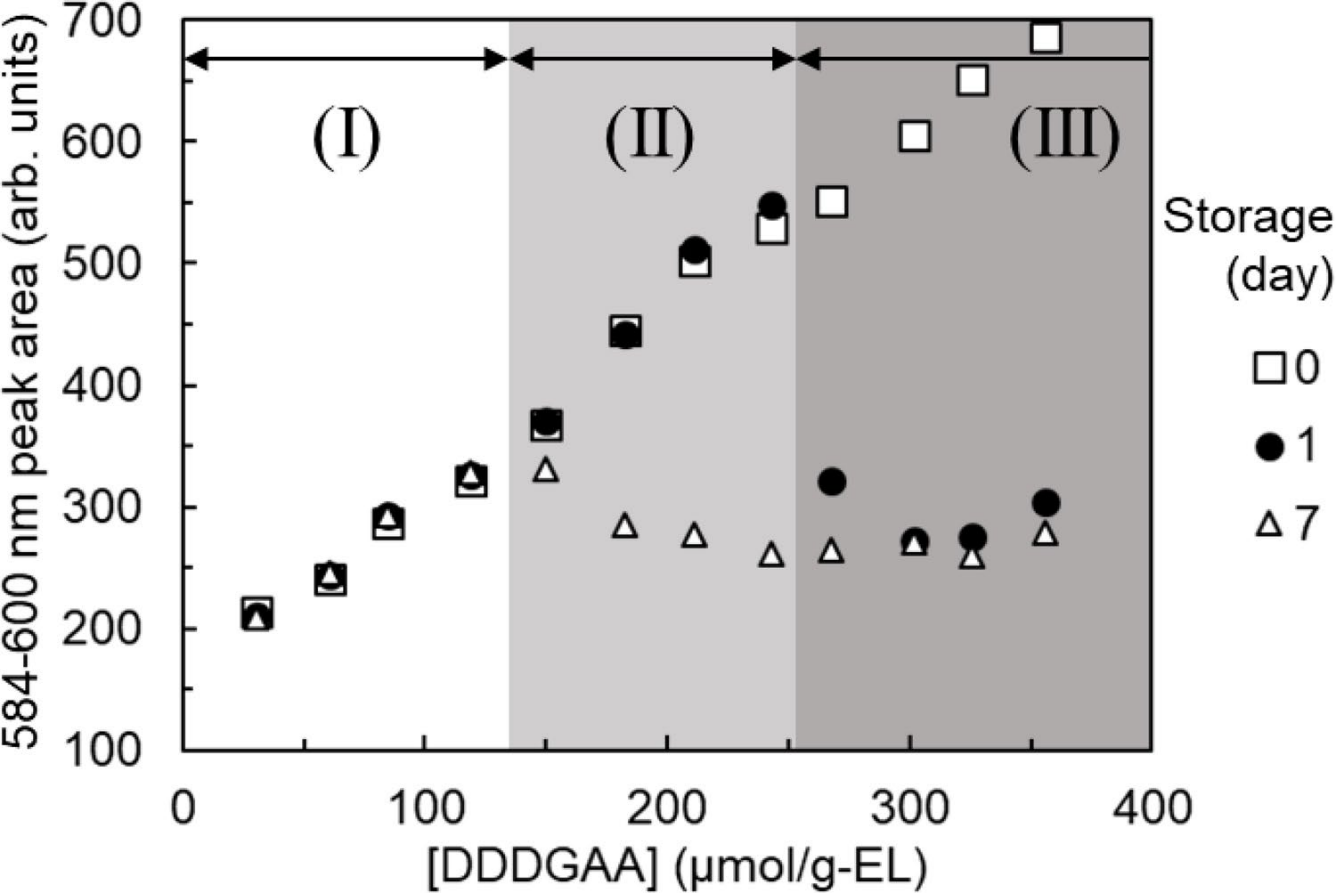
As for Fig. 9, but showing the changes in fluorescence peak area versus DDDGAA concentration

**Fig. 11:**
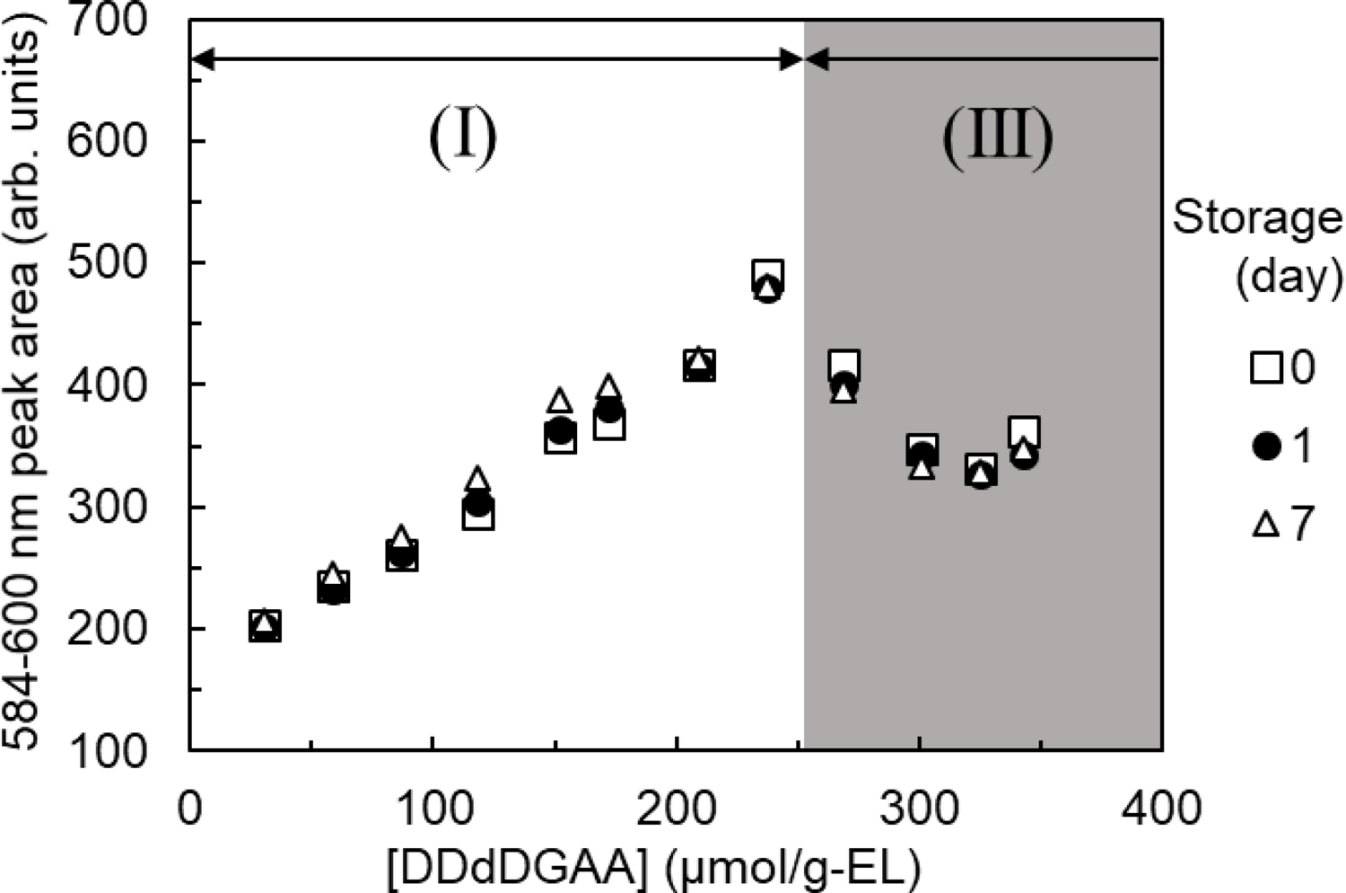
As for Fig. 9, but showing the changes in fluorescence peak area versus DDdDGAA concentration

The Eu adsorption amounts were inferred from the Eu fluorescence peak areas of the DODGAA (Fig. 9), DDDGAA (Fig. 10), and DDdDGAA (Fig. 11) ligands at different concentrations (30–356 µmol/g-EL). In general, the fluorescence peak areas of the freshly prepared liposomes increased with increasing ligand concentration, but this trend was lost in dispersions containing DDdDGAA at concentrations of 268 µmol/g-EL or higher. The cloudiness of the DDdDGAA dispersions did not dissipate even after sonication during the liposome preparation (Fig. S5), indicating that ligand incorporation is limited above ligand concentrations of 268 µmol/g-EL.

The adsorption amounts of liposomes stored for 1 and 7 days decreased at higher ligand concentrations, as evidenced by the appearance of white precipitates (Fig. S6) in some liposome dispersions. The infrared spectra of these precipitates matched that of the DGAA ligand (Fig. S7), suggesting that the DGAA ligand gradually detached from the liposomes and the adsorption capacity diminished accordingly.

Defining a decrease in liposome adsorption capacity as a > 5% reduction in the Eu fluorescence peak area relative to the initial value, the incorporated DGAA ligand concentrations was divided into three regions: the “Long-Term Stability Region” (region I), in which incorporation remained stable for 7 days; the “Short-Term Stability Region” (region II), in which the ligand desorbed between 1 and 7 days, and the “Unstable Region” (region III), in which the ligand desorbed within 1 day or was never incorporated. Table 1 lists the maximum concentrations (the “stable incorporated concentrations”) of the ligands in the long-term stability region. The stable incorporated concentrations increased with increasing length of the alkyl chain, indicating that longer alkyl chains are more stably incorporated than shorter chains.

**Table 1:** Stable incorporated concentrations of the ligands

Ligand	Stable incorporated concentration (µmol/g-EL)
DODGAA	89
DDDGAA	119
DDdDGAA	237

### Discussion and future prospects

The alkyl chain length of the ligand affected the incorporation stability but not the adsorption behavior of the liposome-based extraction system. Increasing the chain length improved the incorporation stability of the ligand but did not significantly alter the adsorption characteristics (preference for heavy rare-earth elements and enhanced adsorption at higher pH), from those of conventional solvent-extraction systems using DGAA [13]. This finding indicates that adsorption behavior is mainly controlled by the ligand head group.

The investigated DGAA ligands exhibited limited adsorption under highly acidic conditions such as those encountered in HLLW streams (3–4 M HNO_3_) [2], so are unsuitable for HLLW separation. Alternative ligands such as *N*,* N*,*N’N’*-tetraoctyldiglycolamide, which demonstrate higher adsorption capacity in strongly acidic environments, may be more appropriate [21]. However, the feasibility of incorporating bulkier ligands within liposomes remains an open question and should be addressed in future work.

## Conclusion

This study investigated the Ln adsorption behavior of an extraction system developed by incorporating DGAA ligands into liposomes. Eu fluorescence measurements demonstrated that the system rapidly reaches equilibrium, with likely interaction of Eu at the liposome surface. The alkyl chain length of the ligand little affected the adsorption behavior but substantially affected the stability of ligand incorporation. Ligands with longer chains were retained over longer times within the liposome than shorter ligands.

Comparing the Ln distribution ratios, the rare-earth selectivity of liposome-incorporated DGAA ligands was deemed similar to that of conventional solvent-extraction systems. Importantly, this behavior was achieved in the absence of organic solvents, highlighting the environmental advantage of the liposome-based approach.

Overall, adsorption is primarily governed by the hydrophilic head group of the ligand, while the alkyl chain length modulates the incorporation stability. Therefore, the liposome-based extraction method provides a flexible platform on which the ligand structure and alkyl chain length can be potentially optimized for adsorbing elements other than Ln. This approach represents a promising new separation technology in analytical chemistry, with potential applications to Ln recovery from HLLW and environmental samples under suitable conditions.

## Supplementary Information

Below is the link to the electronic supplementary material.

Supplementary Material 1

## Data Availability

We do not share the data presented in this paper.

## References

[1] C. Tunsu, M. Petranikova, M. Gergoric, C. Ekberg, T. Retegan, Hydrometallurgy. **156**, 239 (2015)

[2] S.A. Ansari, P. Pathak, P.K. Mohapatra, V.K. Manchanda, Chem. Rev. **112**, 1751 (2012) 22053797 10.1021/cr200002f

[3] M. Salvatores, G. Palmiotti, Prog Part. Nucl. Phys. **66**, 144 (2011)

[4] A. Leoncini, J. Huskens, W. Verboom, Chem. Soc. Rev. **46**, 7229 (2017) 29111553 10.1039/c7cs00574a

[5] M. Hatakeyama, Y. Nishiyama, H. Nagatani, H. Okamura, H. Imura, Solvent Extr. Res. Dev. Jpn. **25**, 79 (2018)

[6] K. Shimojo, K. Kurahashi, H. Naganawa, Dalton Trans. **37**, 5083 (2008) 10.1039/b810277p 18802624

[7] T. Shinozaki, T. Ogata, R. Kakinuma, H. Narita, C. Tokoro, M. Tanaka, Ind. Eng. Chem. Res. **57**, 11424 (2018)

[8] S.A. Ansari, P.K. Mohapatra, J. Chromatogr. A **1499**, 1 (2017) 28408043 10.1016/j.chroma.2017.03.035

[9] A. Miyagawa, T. Takahashi, Y. Kuzure, H. Iwamoto, T. Arai, S. Nagatomo, S. Watanabe, Y. Sano, K. Nakatani, *Anal. Sci.*, 2023, *39*, 1929 10.1007/s44211-023-00402-9 37555916

[10] T.M. Allen, P.R. Culis, Adv. Drug Deliv Rev. **65**, 36 (2013) 23036225 10.1016/j.addr.2012.09.037

[11] S. Orita, S. Shimanuki, S. Okada, K. Nakamura, H. Nakamura, Y. Kitamoto, Y. Shimoyama, Y. Kurashina, Ultrason. Sonochem. **94**, 106326 (2023) 36796146 10.1016/j.ultsonch.2023.106326 PMC9958408

[12] H. Naganawa, K. Shimojo, H. Mitamura, Y. Sugo, J. Noro, M. Goto, Solvent Extr. Res. Dev. Jpn. **14**, 151 (2007)

[13] K. Shimojo, H. Naganawa, J. Noro, F. Kubota, M. Goto, Anal. Sci. **23**, 1427 (2007) 18071230 10.2116/analsci.23.1427

[14] van J.H. Zanten, D.S.-W. Chang, I. Stanish, H.G. Monbouquette, J. Membr. Sci. **99**, 49 (1995)

[15] H.A. Gazzaz, B.H. Robinson, Langmuir. **16**, 8685 (2000)

[16] Y. Sugiura, T. Ishidera, N. Aoyagi, H. Mei, T. Saito, Y. Tachi, Appl. Clay Sci. **258**, 107476 (2024)

[17] T. Ogata, H. Narita, Materials. **17**, 2648 (2024) 38893911 10.3390/ma17112648 PMC11173636

[18] S. Yamasaki, O. Shirai, K. Kano, N. Kozai, F. Sakamoto, T. Ohnuki, Chem. Lett. **42**, 819 (2013)

[19] A.S. Suneesh, K.A. Venkatesan, K.V. Syamala, M.P. Antony, P.R. Vasudeva Rao, Radiochim Acta. **100**, 425 (2012)

[20] P.N. Pathak, S.A. Ansari, S.V. Godbole, A.R. Dhobale, V.K. Manchanda, Spectrochim Acta A **73**, 348 (2009) 10.1016/j.saa.2009.02.040 19329353

[21] M. Husain, S.A. Ansari, P.K. Mohapatra, R.K. Gupta, V.S. Parmar, V.K. Manchanda, Desalination. **229**, 294 (2008)

