# Supplementary material for: Effect of ligand alkyl chain length on lanthanide extraction using liposomes incorporating diglycolamic acid
Source: Anal Sci. 2026 Mar 13;42(4):269–79. doi: 10.1007/s44211-026-00889-y (PMC13013227; doi:10.1007/s44211-026-00889-y)
Supplement: Supplementary file 1 — Supplementary Material 1 [file 44211_2026_889_MOESM1_ESM.docx]

Supplementary Information

**Effect of Ligand Alkyl Chain Length on Lanthanide Extraction Using Liposomes Incorporating Diglycolamic Acid**

Takeru Uehara, Shinya Yamasaki, Sota Shimizu, Yuichi Takaku, Yudai Shigekawa, Aya Sakaguchi

Faculty of Pure and Applied Sciences and Center for Research in Radiation, Isotopes, and Earth System Sciences, University of Tsukuba, 1-1-1 Tennodai, Tsukuba, Ibaraki, 305-8577, Japan

**Corresponding author**: Shinya Yamasaki

**E-mail**: [s-yamasaki@ied.tsukuba.ac.jp](mailto:s-yamasaki@ied.tsukuba.ac.jp)

**Telephone**: +81-29-853-2515

**Present address**: Faculty of Pure and Applied Sciences and Center for Research in Radiation, Isotopes, and Earth System Sciences, University of Tsukuba, 1-1-1 Tennodai, Tsukuba, Ibaraki, 305-8577, Japan

**SI. 1 Synthesis of DDDGAA, DDdDGAA**

*N*,*N*-Didecyldiglycolamic acid (DDDGAA) was synthesized using the following procedure. Diglycolic anhydride (4.0 g, 34 mmol) was dispersed in CH_2_Cl_2_ (20 mL). Didecylamine (5.0 g, 17 mmol) dissolved in CH_2_Cl_2_ (30 mL) was added to the solution. The mixture was stirred at room temperature for 20 hours. The resulting cloudy solution was washed five times with ultrapure water and dried with anhydrous sodium sulfate. The solvent was removed in vacuo to give the crude product, which was recrystallized from n-hexane to obtain DDDGAA as a white powder (3.0 g, 42% yield). ^1^H NMR (600 MHz, CDCl_3_): δ0.86 (t, 6H, N-(CH_2_)_9_-C*H*_3_), 1.23-1.29 (m, 28H, N-(CH_2_)_2_-(C*H*_2_)_7_-CH_3_), 1.53 (qt, 4H, N-CH_2_-C*H*_2_-(CH_2_)_7_-CH_3_), 3.06, 3.33 (t, 4H, N-C*H*_2_-(CH_2_)_8_-CH_3_), 4.19 (s, 2H, N-CO-C*H*_2_-O), 4.37 ppm (s, 2H, C*H*_2_-COOH). EA: Found. C 69.69%, H 11.49%, N 3.48%; Calcd. for C_24_H_47_NO_4_: C 69.69%, H 11.45%, N 3.39%.

**
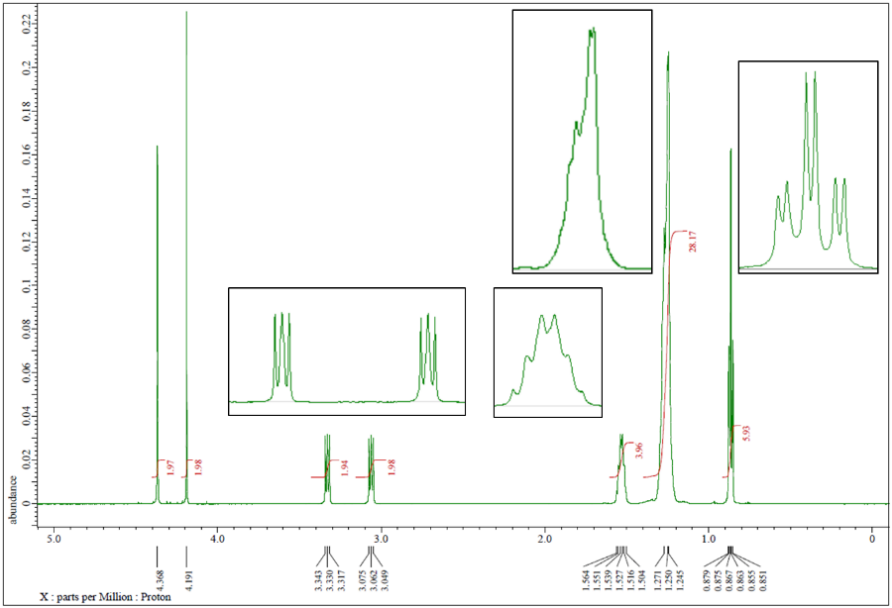
**

*N*,*N*-Didodecyldiglycolamic (DDdDGAA) was synthesized using the following procedure. Diglycolic anhydride (3.0 g, 26 mmol) was dispersed in CH_2_Cl_2_ (20 mL). Didodecylamine (5.0 g, 14 mmol) dissolved in CH_2_Cl_2_ (30 mL) was added to the solution. The mixture was stirred at room temperature for 16 hours. The resulting cloudy solution was washed four times with ultrapure water and dried with anhydrous sodium sulfate. The solvent was removed in vacuo to give the crude product, which was recrystallized from n-hexane to obtain DDdDGAA as a white powder (5.4 g, 81% yield). ^1^H NMR (600 MHz, CDCl_3_): δ0.87 (t, 6H, N-(CH_2_)_11_-C*H*_3_), 1.23-1.29 (m, 36H, N-(CH_2_)_2_-(C*H*_2_)_9_-CH_3_), 1.54 (qt, 4H, N-CH_2_-C*H*_2_-(CH_2_)_9_-CH_3_), 3.06, 3.33 (t, 4H, N-C*H*_2_-(CH_2_)_10_-CH_3_), 4.19 (s, 2H, N-CO-C*H*_2_-O), 4.37 ppm (s, 2H, C*H*_2_-COOH). EA: Found. C 71.70%, H 11.87%, N 3.00%; Calcd. for C_24_H_47_NO_4_: C 71.59%, H 11.80%, N 2.98%.


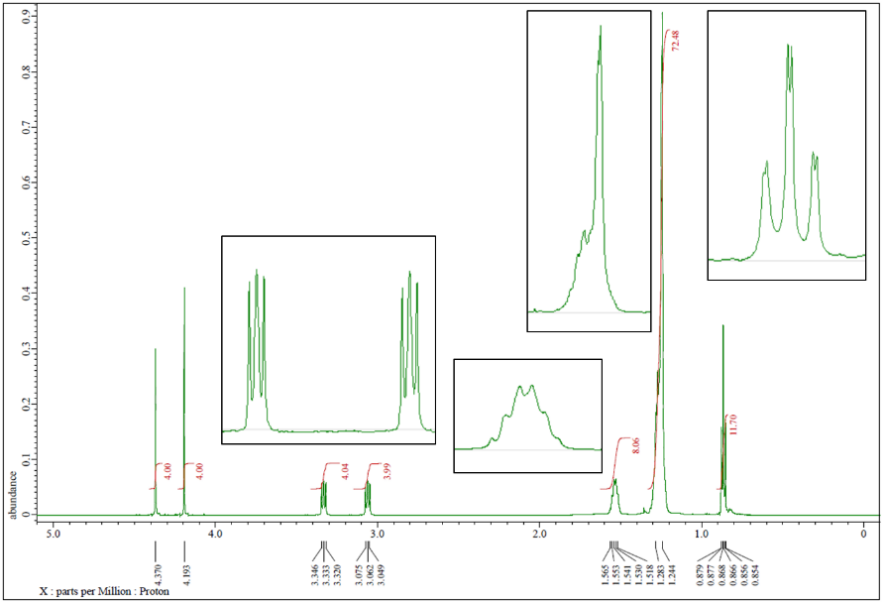


^1^H NMR spectrum was obtained using Nuclear Magnetic Resonance Spectrometer 600 at the Chemical Analysis Division and Open Facility, Research Facility Center for Science and Technology, University of Tsukuba.

The carbon, hydrogen, and nitrogen contents were determined by combustion analysis with a Yanaco MT-6 CHN Corder.

**SI. 2 Supplementary Tables**

**Table 1 Purity of Reagents**

| Reagent | Grade | purity |
| --- | --- | --- |
| Egg Lecithin | For Biochemistry | - |
| *N*,*N*-Dioctyldiglycolamic acid | For laboratory use only | >98.0% |
| Lanthanum(III) nitrate hexahydrate | - | >99.9% |
| Cerium(III) nitrate hexahydrate | High purity reagent | >98.5% |
| Praseodymium(III) nitrate hexahydrate | High purity reagent | >99.95% |
| neodymium(III) nitrate hexahydrate | High purity reagent | >99.95% |
| samarium(III) nitrate hexahydrate | High purity reagent | >99.95% |
| europium(III) nitrate hexahydrate | High purity reagent | >99.95% |
| gadolinium(III) nitrate hexahydrate | High purity reagent | >99.95% |
| terbium(III) nitrate hexahydrate | High purity reagent | >99.95% |
| dysprosium(III) nitrate pentahydrate | High purity reagent | >99.95% |
| lutetium(III) nitrate tetrahydrate | High purity reagent | >99,95% |
| Holmium(III) nitrate hydrate | - | >99.5% |
| erbium(III) nitrate hydrate | - | >99.5% |
| ytterbium(III) nitrate hydrate | - | >99.9% |
| Thulium(III) nitrate | - | 99.9% |

**Table 2 Zeta potential**

| DODGAA conc. | Storage time | Zeta potential | Particle size distribution |
| --- | --- | --- | --- |
| 0 | 1 h | $-14.7\pm0.4 mV$ | Fig. S1 |
| 315 µmol/g-EL | 1 h | $-20.9\pm3.8 mV$ | Fig. S2 |
| 315 µmol/g-EL | 24 h | $-16.0\pm0.1 mV$ | Fig. S3 |

**SI. 3 Supplementary images**


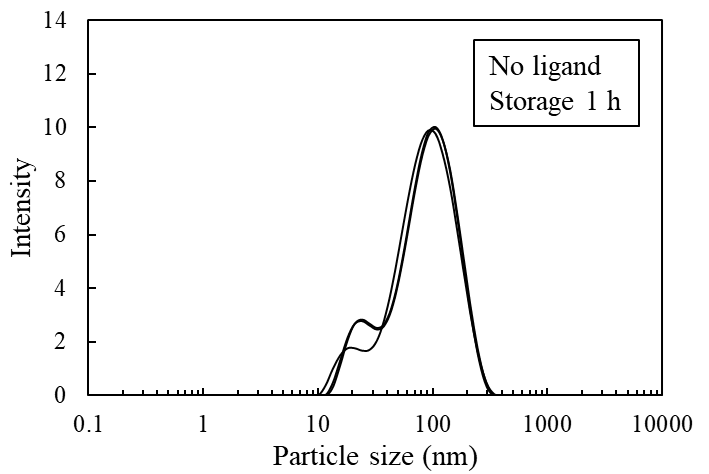


**Fig. S1 Particle size distribution of prepared liposome dispersion (No ligand, EL: 10.0 mg/g, Storage time: 1 h, 0.20 µm filtrated)**


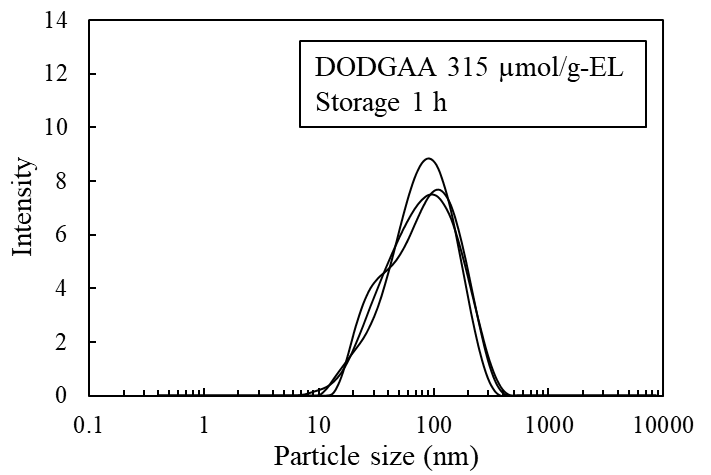


**Fig. S2 Particle size distribution of prepared liposome dispersion (DODGAA: 315 µmol/g-EL, EL: 10.0 mg/g, Storage time: 1 h, 0.20 µm filtrated)**

**
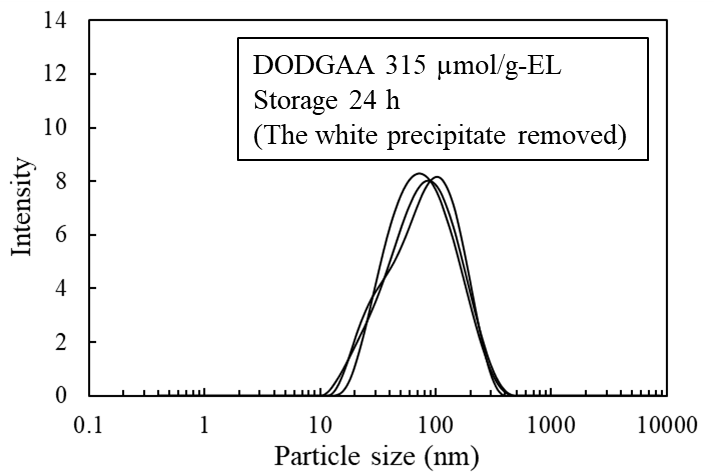
**

**Fig. S3 Particle size distribution of prepared liposome dispersion (DODGAA: 315 µmol/g-EL, EL: 10.0 mg/g, Storage time: 24 h, 0.20 µm filtrated, the white precipitate removed)**


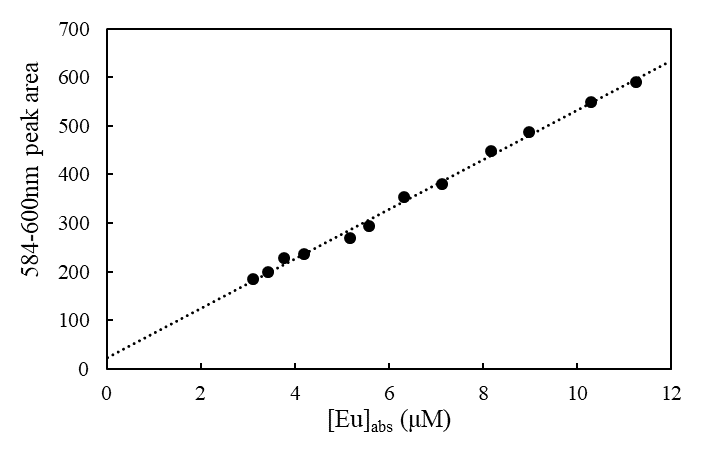


Fig. S4 Correlation between quantification by ICP-MS and Eu fluorescence peak area by fluorescence measurement; correlation coefficient: 0.998.


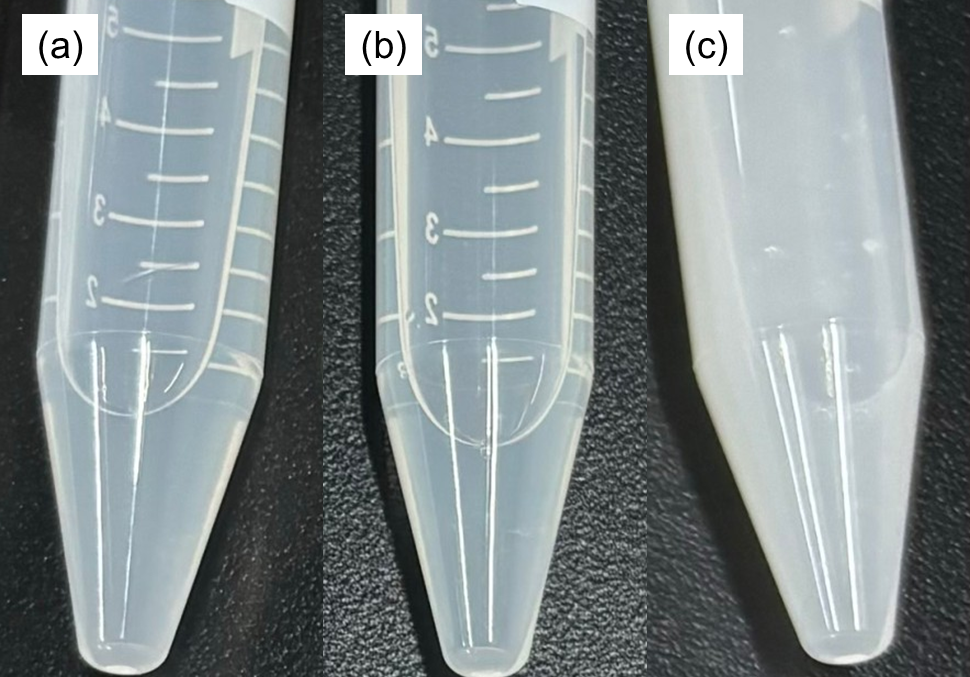


Fig. S5 Difference in appearance of liposome dispersions after sonication depending on DDdDGAA concentration, (a) 30 µmol/g-EL (b) 160 µmol/g-EL (c) 340 µmol/g-EL; [EL]: 10.0 mg/g


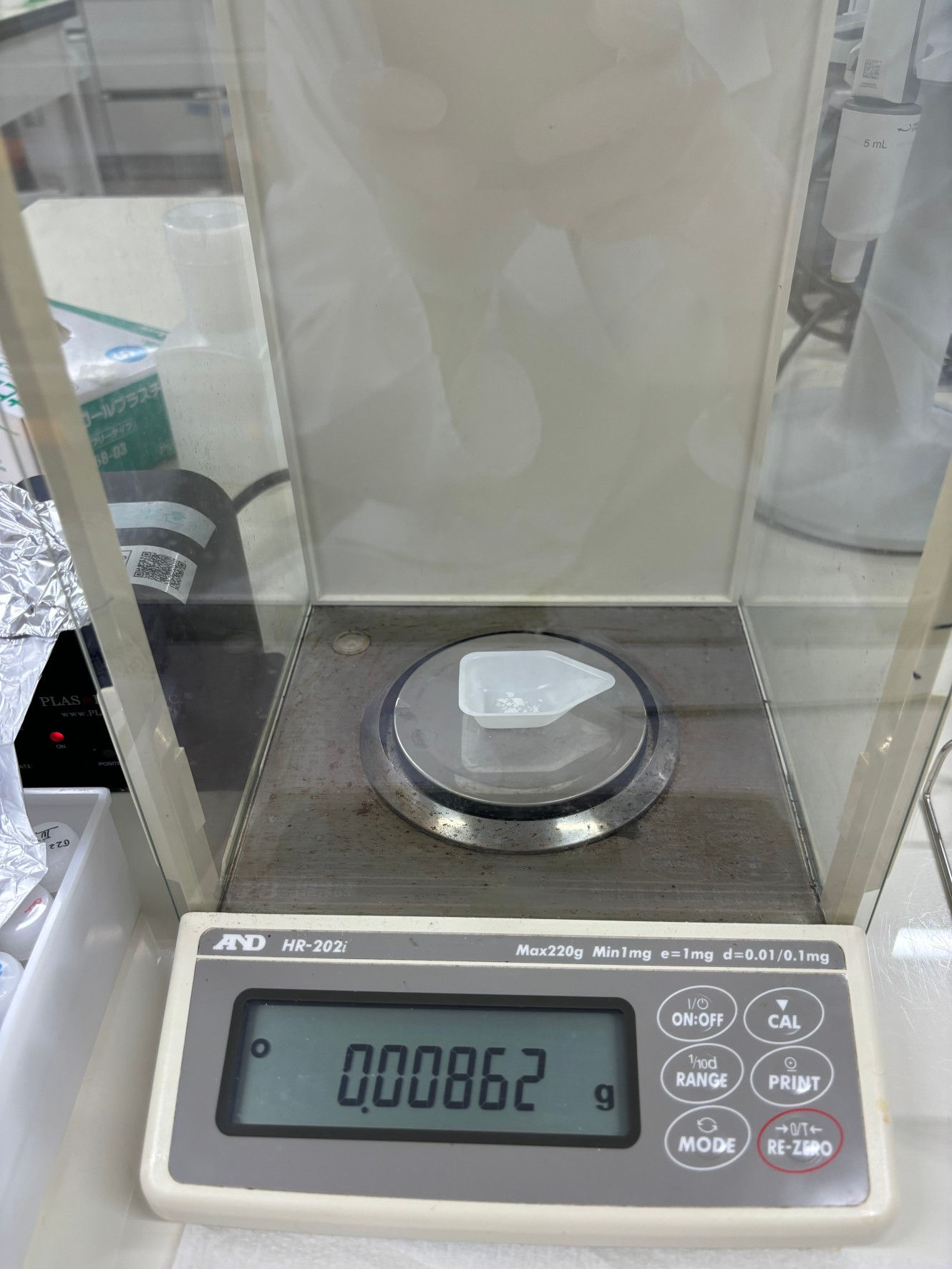


Fig. S6 Photo of white precipitate formed from DODGAA-liposome dispersion; DODGAA: 280 µmol/g-EL; EL: 10.0 mg/g; storage time: 14 days


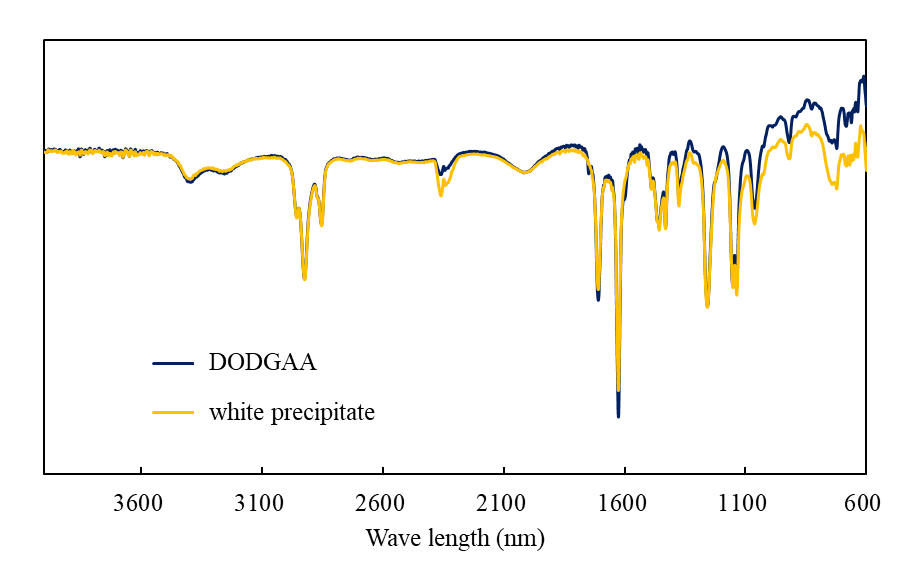


Fig. S7 IR spectrum of DODGAA and white precipitate obtained from DODGAA-liposome solution; DODGAA: 280 µmol/g-EL; EL: 10 mg/g; storage time: 14 days.
